# Large single crystal growth, transport property, and spectroscopic characterizations of three-dimensional Dirac semimetal Cd_3_As_2_

**DOI:** 10.1038/srep12966

**Published:** 2015-08-14

**Authors:** R. Sankar, M. Neupane, S.-Y. Xu, C. J. Butler, I. Zeljkovic, I. Panneer Muthuselvam, F.-T. Huang, S.-T. Guo, Sunil K. Karna, M.-W. Chu, W. L. Lee, M.-T. Lin, R. Jayavel, V. Madhavan, M. Z. Hasan, F. C. Chou

**Affiliations:** 1 Institute of Physics, Academia Sinica, Taipei 11529, Taiwan; 2 Center for Condensed Matter Sciences, National Taiwan University, Taipei 10617, Taiwan; 3 Joseph Henry Laboratory, Department of Physics, Princeton University, Princeton, New Jersey 08544, USA; 4 Department of Physics, National Taiwan University, Taipei 10617, Taiwan; 5 Department of Physics, Boston College, Chestnut Hill, Massachusetts 02467, USA; 6 Institute of Atomic and molecular Sciences, Academia Sinica, Taipei 10617, Taiwan; 7 Anna University, Crystal Growth Centre, Chennai 600025, India; 8 Department of Physics and FrederickSeitz Materials Research Laboratory, University of Illinois Urbana-Champaign, 61801, USA; 9 Princeton Center for Complex Materials, Princeton University, Princeton, New Jersey 08544, USA; 10 National Synchrotron Radiation Research Center, Hsinchu 30076, Taiwan; 11 Taiwan Consortium of Emergent Crystalline Materials, Ministry of Science and Technology, Taipei 10622, Taiwan

## Abstract

The three dimensional (3D) Dirac semimetal is a new quantum state of matter that has attracted much attention recently in physics and material science. Here, we report on the growth of large plate-like single crystals of Cd_3_As_2_ in two major orientations by a self-selecting vapor growth (SSVG) method, and the optimum growth conditions have been experimentally determined. The crystalline imperfections and electrical properties of the crystals were examined with transmission electron microscopy (TEM), scanning tunneling microscopy (STM), and transport property measurements. This SSVG method makes it possible to control the as-grown crystal compositions with excess Cd or As leading to mobilities near 5–10^5^ cm^2^V^−1^s^−1^. Zn-doping can effectively reduce the carrier density to reach the maximum residual resistivity ratio (RRR
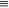
ρ_300K_/ρ_5K_) of 7.6. A vacuum-cleaved single crystal has been investigated using angle-resolved photoemission spectroscopy (ARPES) to reveal a single Dirac cone near the center of the surface Brillouin zone with a binding energy of approximately 200 meV.

Cadmium arsenide (Cd_3_As_2_) is a degenerate n-type semiconductor of the II-V family with high mobility, low effective mass, and a highly non-parabolic conduction band^1^. It exhibits an inverted band structure (optical energy gap E_g_ < 0) comparable to the strained topological insulator HgTe; however, the conduction and valence bands touch at the Dirac nodes in the bulk band structure, giving rise to bulk Dirac fermions featuring robust topologically protected linear dispersion in all three dimensions. These properties are all highly valued for potential applications^2^ and may provide insight into the formation of other exotic phases such as topological superconductors^3^ and Weyl semimetals^4^.

Because of the unusual electrical properties of Cd_3_As_2_^5^,^6^,^7^,^8^, namely the high carrier concentration of 2 × 10^18^ cm^−3^ coupled with a high mobility of >10,000 cm^2^/V-s at room temperature^9^, it has been the subject of many publications concerning its unique transport properties. Additionally, the electron and hole effective masses are 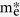 = 0.016 m_o_ and 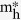 = 0.12 m_o_, giving an estimated exciton Bohr radius of ~47 nm. Thus, this material is expected to show extreme quantum confinement similar to that observed in PbSe (exciton Bohr radius of ∼45 nm)^10^. Several optical and magneto-optical experiments have also been reported^11^,^12^,^13^,^14^. For an adequate description of the energy dependent anisotropy of the cyclotron mass and Shubnikov-de Haas (SdH) oscillation period, Bodnar^15^ proposed a band structure model of Cd_3_As_2_, which is consistent with the available experimental data. However, the results of transport and optical measurements have been interpreted within an isotropic Kane-type band model^16^,^17^.

Topological insulators (TIs) are new quantum materials characterized by a bulk insulating gap and gapless surface states protected by time reversal symmetry, which is realized by spin-orbit coupling induced band inversion with an odd number of Dirac cones^18^,^19^,^20^. Recently, the topological classification of materials has been extended to higher dimension in the so-called three-dimensional topological Dirac semimetal (TDS) phase^21^,^22^. In contrast to TIs, the TDS phase exhibits linear dispersion in all three dimensions and is protected by the crystalline symmetry. A TDS phase was predicted theoretically in Na_3_Bi and Cd_3_As_2_ materials^21^,^22^ and confirmed experimentally using angle-resolved photoemission spectroscopy (ARPES)^23^,^24^. Note that these TDS phases may be a new type of 3D-Dirac semimetal due to the lack of inversion symmetry, which causes the lifting of the spin degeneracy of certain bands in the vicinity of the Dirac point, thereby raising the possibility of realizing the Weyl semimetal phase^21^,^22^.

The growth and characterization of these semimetals has recently generated much attention. Cd_3_As_2_ growth from the vapor phase has been previously reported with limited sizes^25^,^26^,^27^,^28^, including growth under a hydrogen gas flow^29^,^30^. In addition, although Cd_3_As_2_ melts at 721 °C, it undergoes a phase change between 578 °C and 615 °C^29^,^30^,^31^,^32^, and a consequent expansion on cooling. Therefore, growing Cd_3_As_2_ from the vapor phase at a temperature below the solid-solid phase transition temperature is desirable to avoid cracking of the crystals on cooling. In this work, we present a detailed report on the self-selecting vapor growth (SSVG) technique [Fig. 1(a)] of crystal growth and characterization of large-size and high quality single crystals of Cd_3_As_2_. In particular, we have successfully generated crystals containing two types of self-selected large facets, namely the (112) and (even 00) orientations [Fig. 1(b)]. Our study will be useful toward achieving the stable electronic phase and the Weyl semimetal phases in TDS.

## Results and Discussions

The x-ray diffraction patterns were analyzed using the General Structure Analysis System (GSAS) program following the Rietveld profile refining method^33^. Figure 2 shows the x-ray diffraction (XRD) pattern of the as-grown sample and the refined synchrotron x-ray diffraction pattern following the Rietveld profile method. It can be seen that all of the diffraction peaks can be indexed with the I4_1_cd space group which is in agreement with recent reports by H. Yi *et al*.^34^ The Rietveld refinement on the XRD pattern yields lattice constants of *a* = 12.6512(3) Å and *c* = 25.4435(4) Å. The refined structural parameters of the Cd_3_As_2_ with I4_1_cd space group obtained at 300 K by synchrotron x-ray diffraction analysis are summarized in Table 1. In addition, the detailed characterization of Cd_3_As_2_ single crystal with I4_1_/acd space group as described in the supplementary section from Figures S1-S5. The crystal structure of Cd_3_As_2_ has been shown to be complex, depending strongly on the growth temperature and quenching rate^35^. The published phase diagram indicates that more than four different symmetries can be identified as the sample is quenched or slowly cooled from different temperature ranges, from the high symmetry fcc structure of space group Fm 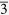
m (No. 225) quenched above 721 °C, to the low symmetry tetragonal structure of space group I4_1_cd (No. 110) after slow cooling. Cd_3_As_2_ can be viewed as a large stacked fluorite-type unit cell with ordered Cd vacancies.

**Figure 1:**
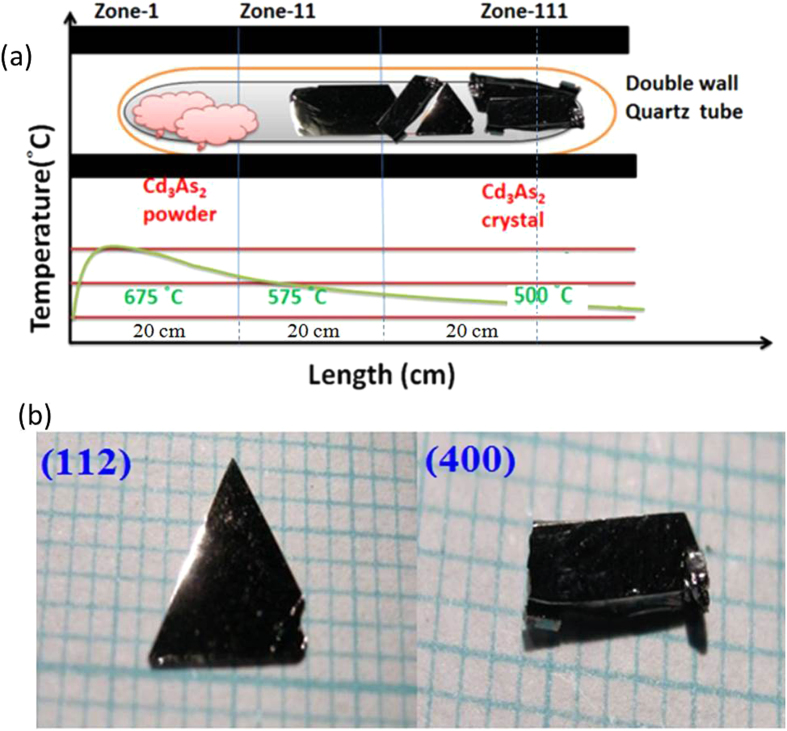
(**a**) Cross-sectional side view of an alumina furnace used for self-selecting vapor growth of a Cd_3_As_2_ single crystal. The positions of the ampoule in which transport takes place and the temperature profile of the furnace for Cd_3_As_2_ growth are shown. (**b**) Cd_3_As_2_ single crystals grown by the self-selecting vapor growth (SSVG) method. The larger facets are (112) and (even 0 0) planes, respectively.

Depending on the growth conditions, there have been two different space groups assigned to the crystals obtained from slow cooling: the noncentrosymmetric I4_1_cd and the I4_1_/acd with centrosymmery^36^,^37^. The I4_1_cd and I4_1_/acd symmetry difference is a reflection of the different ordering types of CdAs_4_ tetrahedral unit packed in a large unit cell of stacked fluorite-like structure with vacancies. The crystal of I4_1_/acd space group has a morphology of needle-shape [Figs S1 and S2] along the <110> direction^37^,^38^, which is grown mostly near the high temperature 575 °C region of sharper thermal gradient in whisker-like growth. As a result, needle crystal of I4_1_/acd symmetry is expected to show much less defect comparing to that of I4_1_cd symmetry with large (112) area of plate-like morphology. On the other hand, because the needle crystal of I4_1_/acd (No. 142) space group has an unexpected high symmetry, even higher than the intermediate temperature quenched phase of P4_2_/nmc (No. 137)^37^, the unique morphology of needle growth could be responsible for the sustaining I4_1_/acd space group with centrosymmetry. The stable growth of large crystal of space group I4_1_cd allows the plate-like growth morphology [Fig. 1(b)] of (112) plane as a result of preferable pseudo-hexagonal close packing when c ~ 2a, which could be the reason why noncentrosymmetry is tolerated. The Cd_3_As_2_ crystal structure studied in this report can be satisfactorily indexed with the I4_1_cd space group. Figure 3 shows the I4_1_cd crystal structure with two different planes highlighted in red. The (112) plane, shown in panel (a), exhibits pseudo-hexagonal close-packing because *c* ~ *2a* in the large tetragonal unit cell, and the (even 0 0) plane is shown in rectangular shape in panel (b).

Figure 4(a–c) shows a series of selected area electron diffraction (SAED) patterns taken on a Cd_3_As_2_ single crystal. Tetragonal lattice of space group I4_1_cd is used to index all diffraction spots in Fig. 4a. The observation of (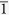
10) spots (yellow circle in Fig. 4a), which are symmetry-forbidden reflections to the I4_1_/acd (extinction condition of *hk*0: *h* and *k* both even) space group, gives a direct evidence for the satisfactory assignment of I4_1_cd space group. Notably, Cd_3_As_2_ was sensitive to the beam irradiation and all patterns were taken with only a relatively low exposure to the electron beam.

ARPES measurements for the low-energy electronic structure investigation were performed at Beam Line 4.0.3 at the Advanced Light Source in Berkeley, California, which is equipped with a high-efficiency R8000 electron analyzer. The energy and momentum resolutions were better than 40 meV and 1% of the surface BZ, respectively. The samples were cleaved *in situ* and measured at 10–80 K in a vacuum better than 10^−10^ torr. The crystals were found to be very stable and exhibited no degradation for the typical measurement period of 20 h. Figure 5(a) shows the core level spectroscopic measurement of the Cd_3_As_2_ sample. Sharp XPS peaks at binding energies of E_B_ ~ 11 eV and 41 eV that correspond to the cadmium 4*d* and the arsenic 3*d* core levels were observed, confirming the correct chemical composition of our samples. Figure 5(b) shows an ARPES dispersion map in the 800 meV binding energy window, where the dispersion of several bands can be identified. Remarkably, a low-lying small feature that crosses the Fermi level is observed, which corresponds to the surface bands of Cd_3_As_2_^22^,^23^. It is important to note that the linearly dispersive upper Dirac cone is located at the surface Brillouin zone center and that the Dirac point is found at a binding energy of 200 meV, consistent with earlier reports^23^,^39^,^40^. Our spectroscopic sample characterization shows a linear dispersive band, thus confirming the high quality of the sample used in our measurements.

STM topographies acquired from the vacuum-cleaved Cd_3_As_2_ surface exhibit a clean step-terrace morphology, as demonstrated in Fig. 6(a). The corresponding height profile is shown in Fig. 6(b). The zoomed-in image on one of the terraces, shown in Fig. 6(c), reveals an atomic surface lattice, which has a clear surface reconstruction^37^,^38^ as shown by the 2D Fourier transform displayed in Fig. 6(d), consistent with that observed by Jeon *et al*.^38^. The observed pseudo-hexagonal nearest-neighbor lattice spacing of d_n-n_ = 0.435 (±0.02) nm is close to that expected for the (112) surface. With this in mind, we interpret the step-height of 0.718 nm observed in the large-scale topography map as corresponding to approximately twice the As-As interlayer spacing perpendicular to the (112) plane (two times √(2/3) x d_n-n_). Tunneling spectroscopy was subsequently performed on this (112) surface, as shown in Fig. 6(e). The results reveal a conductance minimum at approximately 200 meV below the Fermi level, consistent with the energy of the Dirac point observed in the ARPES data. The approximately linear increase in conductance above this point is also consistent with the linearly dispersive upper Dirac cone observed using ARPES.

The resistivity data were measured using a standard four-probe method. The temperature dependence of the resistivity is shown in Fig. 7(a), which shows that metallic behavior is observed in both the pure and doped Cd_3_As_2_ crystals under investigation. The resistivity is nearly temperature independent below T = 5 K, giving a residual resistivity in the range of approximately 0.2–0.4 mΩ-cm. The Hall resistivity is practically linear under a magnetic field of up to 15 Tesla, as shown in Fig. 7(b), with the Shubnikov-de Haas oscillation appearing in higher fields. From the Hall data at T = 2 K, the carrier density equals n = 1.4 × 10^18^cm^−3^ in Cd_2.9_Zn_0.1_As_2_ giving a Hall mobility of μ = 10^5^ cm^2^/V-s. The 10^5^ order of mobility in Cd_3_As_2_ has also been reported recently by Liang *et al*.^41^,^42^,^43^ For pure Cd_3_As_2_ and Cd_2.9_Sn_0.1_As_2_, the carrier densities are higher in the range of n = 2.6–5.2 × 10^18^ cm^−3^. We note that Zn-doping can effectively reduce the carrier density in Cd_3_As_2_, which gives the lowest carrier density and the highest residual resistivity ratio (RRR
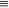
ρ_300K_/ρ_5K_) of approximately 7.6 among the crystals we have studied.

## Conclusions

In summary, large plate-like high quality single crystals of Cd_3_As_2_ with large facets of (112) and (even 0 0) planes have been grown by a self-selecting vapor growth (SSVG) method. The observation of sharp and clean diffraction spots in the SAED patterns indicate the good crystallinity of the single crystals grown, and the symmetry is indexed satisfactorily with the I4_1_cd space group. Tunneling spectroscopy measurements performed using STM on the cleaved (112) surface confirms a conductance minimum at approximately 200 meV below the Fermi level. This is consistent with the energy of the Dirac point, as observed using ARPES. The approximately linear increase in the conductance above this point is also consistent with the linearly dispersive upper Dirac cone observed using ARPES. Cd_3_As_2_ single crystals show a large resistivity anisotropy in the different planes. Zn-doping can effectively reduce the carrier density of Cd_3_As_2_, which gives the lowest carrier density and the highest residual resistivity ratio (RRR 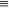 ρ_300K_/ρ_5K_) of approximately 7.6 among the crystals we have studied.

## Experimental Section

### Self- selecting vapor growth of Cd_3_As_2_ single crystals

We have applied self-selecting vapor growth (SSVG) method on the growth of Cd_3_As_2_ single crystals. In this SSVG technique, there is no transport agent used. A selected single crystal orientation can be grown using this SSVG technique. To prepare the source materials, the binary compounds were first synthesized by reacting the 99.9999% pure elemental forms of Cd and As. The nominally stoichiometric proportions were sealed in evacuated ampoules, heated to 50 °C above the melting points of the corresponding compounds for 4 hours, and quenched in water. The pure Cd_3_As_2_ and Sn/Zn-doped Cd_3_As_2_ were purified by an evacuated closed-tube sublimation process, in which sample ingots were held at a temperature near 800 °C to allow vapor deposition on the inner wall of the cold end extending out of the tube furnace.

The next step in preparing the growth precursors is to refine the binary compounds with small amounts of excess metal or chalcogen elements in another evacuated closed ampoule. The precursors were melted at 50 °C above the liquidus temperature for 4 hours and then water-quenched. The quenched ingot was iso-thermally annealed at 700 °C for 3 days and then air-cooled. As illustrated in Fig. 1(a), the SSVG technique requires the heating of a sealed evacuated double quartz ampoule with an outer tube (30 cm length × 1.8 cm inner diameter) and an inner tube (20 cm length × 1.4 cm inner diameter) that contains approximately 20 g of uniformly distributed coarsely powdered source material in a horizontal three-zone furnace (100 cm long × 15 cm bore diameter). Before the ampoule is loaded, it is degreased and etched with aqua regia, rinsed, and heated overnight at 1,000 °C under high vacuum. The furnace is heated to the growth temperature with the designated temperature profile before inserting the ampoule. The growth ampoule rests on the furnace core and near the center of the furnace. The horizontal and vertical temperature gradients measured in the furnace without the ampoule in place are ~0.1–0.2 °C/cm and 1.0–1.4 °C/cm, respectively.

**Figure 2:**
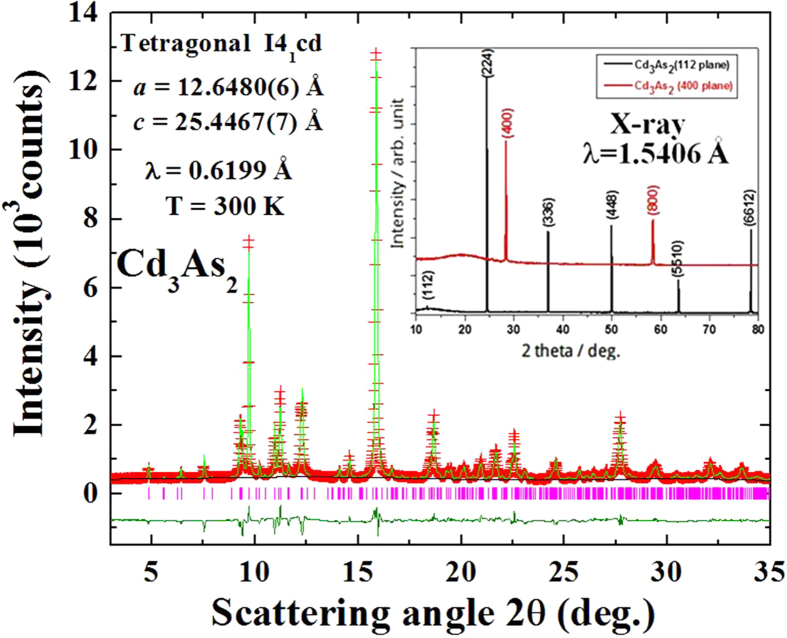
Powder X-ray diffraction pattern of the Cd_3_As_2_ sample (red crosses) and its Rietveld refinement (green curve). The inset shows the obtained diffraction patterns of single crystals with facets of preferred orientations along the <112> and <even 0 0 > directions.

**Figure 3:**
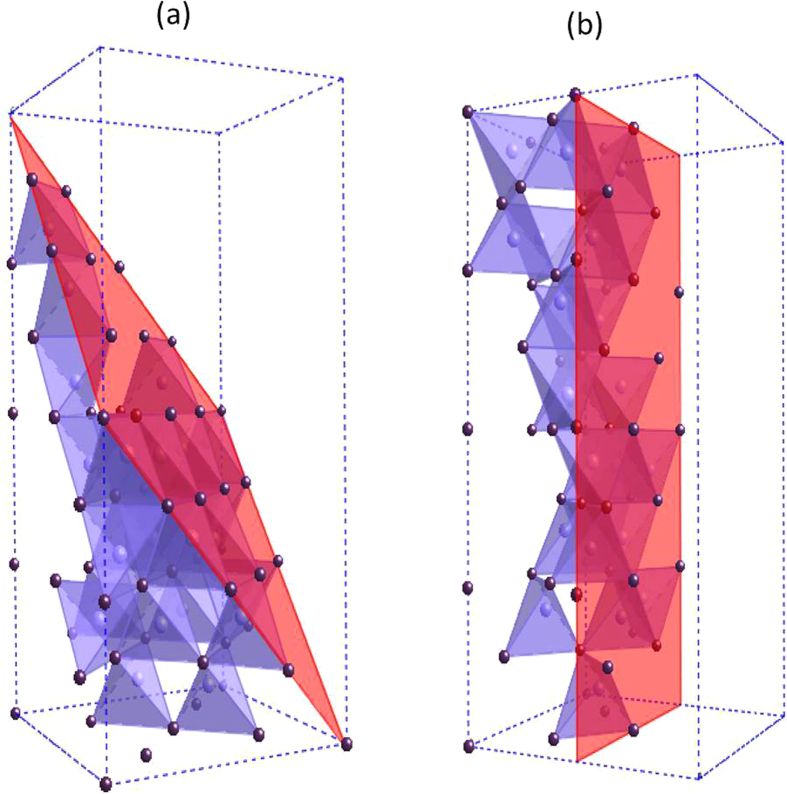
An illustration of the Cd_3_As_2_ crystal structure belonging to the noncentrosymmetric space group I4_1_cd. (**a**) The (112) plane exhibits pseudo-hexagonal close-packing because c ~ 2a in the large tetragonal unit cell. (**b**) The (even 0 0) plane is shown in rectangular shape.

**Figure 4:**
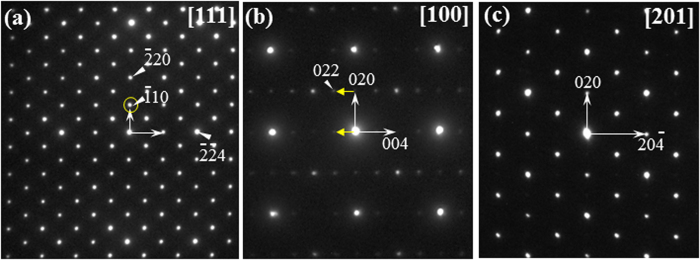
The SAED patterns of the Cd_3_As_2_ crystal taken along the zone axes of (**a**) [111], (**b**) [100], and (**c**) [201] indexed with the I4_1_cd space group. Note that the appearance of symmetry-forbidden spots, 00*l* with *l* = 2n (n odd) in Fig. 4b can be understood as a result of electron multiple scattering. The yellow arrows denote the multiple scattering paths for the (00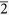) reflection.

**Figure 5:**
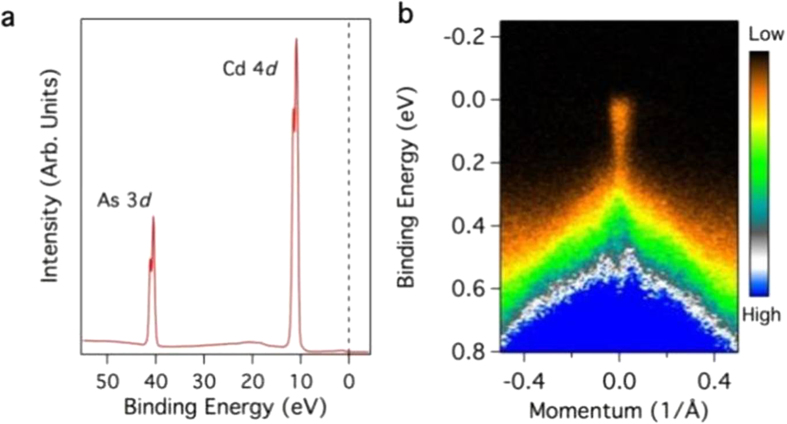
(**a**) Core-level spectroscopic measurement of Cd_3_As_2_, in which the Cd 4*d* and As 3*d* peaks are observed. (**b**) ARPES dispersion map of Cd_3_As_2_ measured near the center of the surface Brillouin zone. The measurements were acquired using a photon energy of 102 eV and at a temperature of 10 K at ALS BL4.

**Figure 6:**
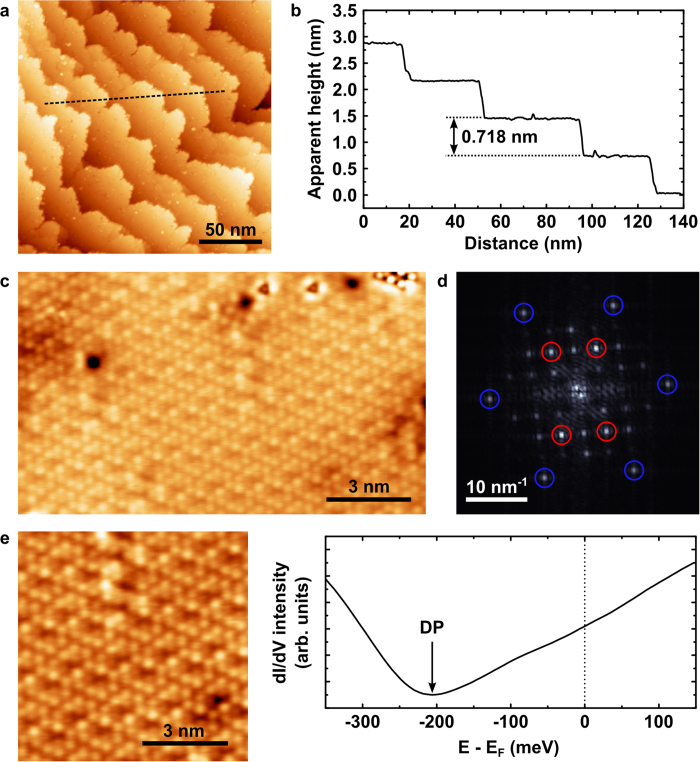
STM topography measurements performed on the cleaved Cd_3_As_2_ (112) surface. (**a**) Large-scale topography map (V_bias_ = 2 V, Iset = 0.1 nA) showing a step-terrace morphology and (**b**) the topographic profile, taken along the dashed line shown in panel (**a**) with a step height of 0.718 nm, corresponding to approximately twice the As-As inter-layer distance perpendicular to the (112) plane. (**c**) Atomically resolved topography (V_bias_ = −100 mV, I_set_ = 1 nA) taken on a terrace, revealing a clear surface re-construction and (**d**) the corresponding 2D-FFT pattern showing peaks for the atomic surface lattice (circled in blue) of nearest- neighbor distance 0.435 (±0.02) nm and for the reconstruction (circled in red). (**e**) Tunneling spectroscopy measurements on the cleaved Cd_3_As_2_ (112) surface. An averaged dI/dV spectrum was acquired with a set-point of V_bias_ = −100 mV, I_set_ = 1 nA from the area shown in the topography image on the left (also taken with V_bias_ = −100 mV, I_set_ = 1 nA). The minimum in the dI/dV curve corresponds to the Dirac point (DP).

**Figure 7:**
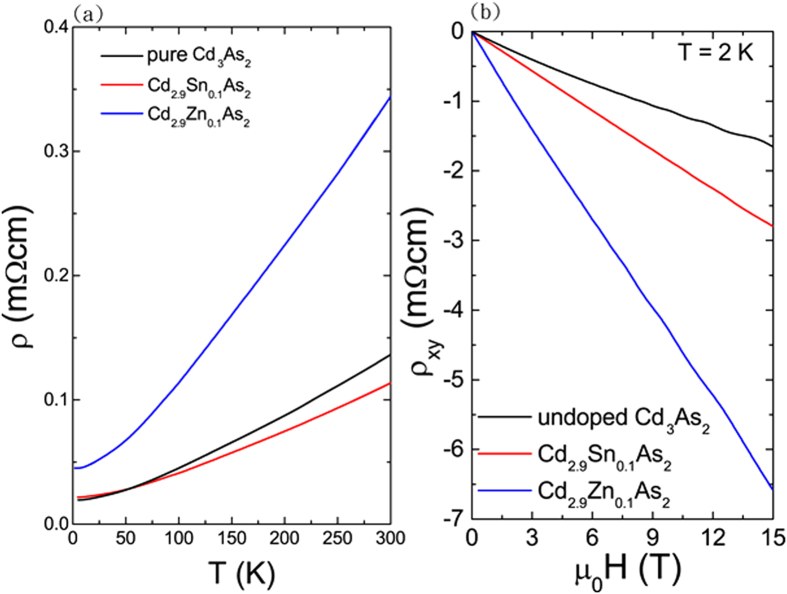
(**a**) Temperature dependence of the resistivity in pure and doped Cd_3_As_2_. (**b**) The corresponding Hall resistivity in pure and doped Cd_3_As_2_.

In the final step of the SSVG method, the ingot was crushed with an agate mortar and pestle, sieved to obtain the proper range of particle sizes (0.1–0. 3 mm diameter), and placed in the growth ampoule. The growth temperature profiles used were approximately 675–575–500 °C for Cd_3_As_2_ and 700–650–600 °C for Sn/Zn-doped Cd_3_As_2_, respectively. The typical growth time used was 10 days. It was found that longer growth time had little effect on both the number and size of the crystals. Crystals with lower dislocation densities can be obtained by leaving the ampoule in the furnace and turning off the power directly. A successful growth exhibits few well separated plate like crystals, as displayed in Fig. 2. These crystals have individual facets of up to 0.7 cm^2^ in area. The facets are typically (112) oriented, although occasionally crystals with large facets of (even 0 0) orientation are present in the middle region. No obvious crystal morphology change in Cd_3_As_2_ is detected with the addition of Zn or Sn.

### Material Characterization

Initial phase characterization was carried out using Bruker-AXS D8 ADVANCE x-ray diffractometer that is equipped with a diffracted beam monochromator set for Cu K_α1_ radiation (λ = 1.54056 Å), and further crystal structure analysis was conducted using synchrotron x-ray powder diffraction (SXRD). The SXRD patterns were collected at room temperature with crushed crystals at the beam line in the National Synchrotron Radiation Research Center (NSRRC), Taiwan with energy of 20 keV, which corresponds to a wavelength of 0.6199 Å. The structural refinement was performed using data obtained from the synchrotron X-ray source facility in NSRRC-Taiwan. The transport properties for the Cd_3_As_2_ crystals were measured using a four-probe method for the in-plane resistivity as a function of temperature. Two types of specimens with crystalline (112) and (even 00) planes were prepared for investigation. The structural characteristics of Cd_3_As_2_ in three-dimensional reciprocal space were also investigated by electron diffraction using a transmission electron microscope TEM (JEOL 2000FX) operated at 200 kV. The electron diffraction measurements were performed on thin regions of freshly crushed single crystals dispersed in a Cu grid. Cleavage of Cd_3_As_2_ crystals for investigation by STM was performed in a preparation chamber with a base pressure lower than 5 × 10^−11^ mbar. The crystals were cleaved on a L-He cooled cryostat, allowing for a cleavage temperature of approximately 8 K. All of the STM measurements were performed at 4.5 K in an Omicron low temperature STM using a chemically etched tungsten tip. dI/dV(V) spectroscopy was performed using a lock-in amplifier with a bias modulation of 30 mV at a frequency of 5.9 kHz.

## Additional Information

**How to cite this article**: Sankar, R. *et al*. Large single crystal growth, transport property, and spectroscopic characterizations of three-dimensional Dirac semimetal Cd_3_As_2_. *Sci. Rep*. **5**, 12966; doi: 10.1038/srep12966 (2015).

## Supplementary Material

Supplementary Information

## Figures and Tables

**Table 1:** Lists of refined structural parameters of the Cd_3_As_2_ sample at 300 K, where B_iso_ represents the isotropic temperature parameter.

Cd_3_As_2_Tetragonal I4_1_cd space group (No. 110, Z = 32) T = 300 K, *a* = *b* = 12.6512(3) Å and *c* = 25.4435(4) Å						
Atom	x	y	z	Site	B_iso_(Å^2^)	Occupancy
Cd1	0.3557	0.1205	0.0613	*16b*	2.41 (5)	1.000
Cd2	0.1183	0.3581	0.0524	*16b*	1.78 (3)	0.996 (2)
Cd3	0.3934	0.3934	0.0726	*16b*	2.29 (4)	1.002 (1)
Cd4	0.1089	0.1057	0.1774	*16b*	2.15 (2)	1.005 (2)
Cd5	0.3795	0.1494	0.1896	*16b*	1.89 (3)	0.997 (3)
Cd6	0.1414	0.3795	0.1966	*16b*	2.14 (2)	1.001 (2)
As1	0	0	0	*8a*	1.77 (4)	0.997 (3)
As2	0	0	0.2495	*8a*	2.04 (3)	1.003 (4)
As3	0.0074	0.2548	0.1236	*16b*	2.36 (4)	0.999 (2)
As4	0.248	−0.0059	0.1254	*16b*	1.71 (5)	1.004 (2)
As5	0.2586	0.2395	0	*16b*	2.22 (3)	0.998 (3)

χ^2^ = 5.854, R_p_ = 6.92%, R_wp_ = 8.12%.

## References

[b1] Sieranski K., Szatkowsky J. & Misievicz J. Semiempirical tight-binding band structure of II3V2 semiconductors: Cd_3_P_2_, Zn_3_P_2_, Cd_3_As_2_, and Zn_3_As_2_. Phys. Rev. B 50, 7331 (1994). 10.1103/physrevb.50.7331 9974709

[b2] Kharzeev D. E. & Yee H.-U. Anomaly induced chiral magnetic current in a Weyl semimetal: Chiral electronics. Phys. Rev. B 88, 115119 (2013).

[b3] Wang H. . Observation of superconductivity in 3D Dirac semimetal Cd_3_As_2_ crystal. *arXiv* 1501. 00418.

[b4] Qi X.-L. & Zhang S.-C. Topological insulators and superconductors. Rev. Mod. Phys. 83, 1057 (2011).

[b5] Zdanowicz W. Electric Properties of Copper-doped Cadmium Arsenide. Acta Phys. Pol. 21, 541 (1962).

[b6] Rosenman I. Quantum transport effects in cadmium arsenide, effective mass tensor and band structure. Phys. Lett. 21, 148 (1966).

[b7] Aubin M., Brizardand R. & Messa J. P. Une deuxième bande de conduction dans Cd_3_As_2_. Can. J.Phys. 48 (19), 2215 (1970).

[b8] Blom F. A. P. & Gelten M. J. Proceedings of the International Conference on the Physics of Marrow Gap Semiconductors, Warsaw, p. 257 (1977). Polish Scientific Publishers (1978).

[b9] Dowgiazzo-Plenkiewicz B. & Plenkiewicz P. Inverted band structure of Cd_3_As_2_. Phys. Status Solidi (b) 94, K57 (1979).

[b10] Wise F. W. Lead Salt Quantum Dots: the Limit of Strong Quantum Confinement. Acc. Chem. Res. 33, 773 (2000). 11087314 10.1021/ar970220q

[b11] Turner W. J., Fischler A. S. & Reese W. E. Electrical and Optical Properties of the II–V Compounds. J. Appl. Phys. 32, 2241 (1961).

[b12] Turner W. J., Fischler A. S. & Reese. Physical properties of several II-V semiconductors. Phys. Rev. 121, 759 (1961).

[b13] Wagner R. J., Palik E. D. & Swiggard E. M. Interband Magneto absorption in Cd_x_Zn_3-x_As_2_ and Cd_3_As_x_P_2-x_. J. Phys. Chem. Solids, Suppl. 1, 471 (1971).

[b14] Aubin M. J. & Cloutier J. P. La thermoréflectance des alliages Cd_3−x_Zn_x_As_2_. Can. J. Phys. 53, 1642 (1975).

[b15] Blom F. A. P. Narrow Gap Semiconductors Physics and Applications. Proceedings of the International Summer School Held in Nimes, France, September 3-15, 1979. Lectures Notes in Physics 133, 191 (1980).

[b16] Blom F. A. P. & Gelten M. J. Temperature dependence of electron concentration in cadmium arsenide. Phys. Rev. B 19, 2411 (1979).

[b17] Gelten M. J., Van Es C. M., Blom F. A. P. & Jonganeelen J. W. F. Optical verification of the valence band structure of cadmium arsenide. Solid State Commun. 33, 833 (1980).

[b18] Hasan M. Z. & Kane C. L. Colloquium: Topological insulators. Rev. Mod. Phys. 82, 3045 (2010).

[b19] Qi X. L. & Zhang S.-C. Topological insulators and superconductors. Rev. Mod. Phys. 83, 1057 (2011).

[b20] Moore J. E. The birth of topological insulators. Nature 464, 194 (2010). 20220837 10.1038/nature08916

[b21] Wang Z. . Dirac semimetal and topological phase transitions in A_3_Bi (A = Na, K, Rb). Phys. Rev. B, 85, 195320 (2012).

[b22] Wang Z., Weng H., Wu Q., Dai Xi & Fang Z. Three-dimensional Dirac semimetal and quantum transport in Cd_3_As_2_. Phy. Rev. B 88, 125427 (2013).

[b23] Neupane M. . Observation of a three-dimensional topological Dirac semimetal phase in high-mobility Cd_3_As_2_. Nat. Commun. 5, 4786 (2014). 24807399 10.1038/ncomms4786

[b24] Liu Z. K. . Discovery of a Three-Dimensional Topological Dirac Semimetal, Na_3_Bi. Science 343, 864 (2014). 24436183 10.1126/science.1245085

[b25] Koltirine B. & Chaumereuil M. Formation de monocristaux d’arseniure de cadmium. Phys. Stat. Sol. 13, K1 (1966).

[b26] Sexer M. J. Sur quelques propriétés de Cd_3_As_2_. Phys. Radium 22, 807 (1961).

[b27] Rosenman I. Effet Shubnikov de Haas dans Cd_3_As_2_: Forme de la surface de Fermi et modele non parabolique de la bande de conduction. J. Phys. Chem. Solids 30, 1385 (1969).

[b28] Hrubý A. & Petrová J. Preparation of Cd_3_As_2_ and CdAs_2_ crystals by transport reaction in vapour phase. Czech. J. Phys. B 21, 890 (1971).

[b29] Sillvey G. A., Lyons V. J. & Silvestri V. J. The Preparation and Properties of Some II – V Semiconducting Compounds. J. Electrochem. Soc. 108, 653 (1961).

[b30] Fiansen M. & Anderko K. Constitution of the Binary Alloys, McGraw-Hill, Maidenhead, p. 157 (1958).

[b31] Jayaraman A., Aanatharaman T. & Klement W. Melting and polymorphism of Zn_3_As_2_ and Cd_3_As_2_ at high pressures. J. Phys. Chem. Solids 27, 1605 (1966).

[b32] Hiscocks S. E. R. The Cd_3_As_2_-NiAs pseudobinary eutectic. J. Mater. Sci. 4, 773 (1969).

[b33] Larson A. C. & Von Dreele A. C. Los Alamos National Laboratory Report No. LAUR086-748 (2000).

[b34] Yi H. . Evidence of Topological Surface State in Three-Dimensional Dirac Semimetal Cd_3_As_2_. Sci. Rep. 4, 6106 (2014). 25139455 10.1038/srep06106 PMC4138522

[b35] Arushanov E. K. II_3_V_2_ compounds and alloys. Prog. Cryst. Growth Charact. Mater. 25, 131 (1992).

[b36] Steigmann G. & Goodyear J. The crystal structure of Cd_3_As_2_. Acta Crystallogr.,Sect. B: Struct. Crystallogr. Cryst. Chem. 24, 1062 (1968).

[b37] Ali M. N. . The Crystal and Electronic Structures of Cd_3_As_2_, the Three-Dimensional Electronic Analogue of Graphene. Inorganic Chemistry 53(8), 4062 (2014). 24679042 10.1021/ic403163d

[b38] Jeon S. . Landau quantization and quasiparticle interference in the three-dimensional Dirac semimetal Cd_3_As_2_. Nature Materials 13, 851 (2014). 24974888 10.1038/nmat4023

[b39] Liu Z. K. . A stable three-dimensional topological Dirac semimetal Cd_3_As_2_. Nature Materials 13, 677 (2014). 24859642 10.1038/nmat3990

[b40] Borisenko S. . Experimental Realization of a Three-Dimensional Dirac Semimetal. Phys. Rev. Lett. 113, 027603 (2014). 25062235 10.1103/PhysRevLett.113.027603

[b41] Liang T. . Ultrahigh mobility and giant magnetoresistance in the Dirac semimetal Cd_3_As_2_. Nat. Mater. 10.1038/ nmat 4143 (2014). 10.1038/nmat4143 25419815

[b42] He L. P. . Quantum Transport Evidence for the Three-Dimensional Dirac Semimetal Phase in Cd_3_As_2_. Phys. Rev. Lett. 113, 246402 (2014). 25541783 10.1103/PhysRevLett.113.246402

[b43] Zhao Y. . Anisotropic Fermi Surface and Quantum Limit Transport in High Mobility 3D Dirac Semimetal Cd_3_As_2_. *arXiv*:1412.0330.

