# Supplementary material for: Large single crystal growth, transport property, and spectroscopic characterizations of three-dimensional Dirac semimetal Cd3As2
Source: Sci Rep. 2015 Aug 14;5:12966. doi: 10.1038/srep12966 (PMC4642520; doi:10.1038/srep12966)
Supplement: Supplementary Information [file srep12966-s1.pdf]

Supplementary

Large single crystal growth, transport property, and spectroscopic characterizations of three-dimensional Dirac semimetal Cd_3As_2

R. Sankar,[a,*] M. Neupane,[b] S.-Y. Xu,[b] C. J. Butler,[c] I. Zejikovic,[d] I. Panneer Muthuselvam,[a,] F.-T. Huang,[a] S.-T.Guo,[e] Sunil K. Karna,[a] M.-W. Chu,[a] W. L. Lee,[e] M.-T. Lin,[c,f] R. Jayavel,[g] V. Madhavan,[h] M. Z. Hasan,[b,I] and F.C.Chou[a,j,k]

Cd_3As_2 of centosymmetric *I*4_1*/acd* space group has a distorted superstructure of the antifluorite structure type, with a tetragonal unit cell of *a* = 12.6567(5) Å and *c* = 25.4562(12) Å [Figure S1]. Figure S2 shows the refined synchrotron x-ray diffraction pattern of Cd_3As_2 sample following the Rietveld profile method. It can be seen that all of the diffraction peaks can be indexed with a centrosymmetric *I*4_1*/acd* space group. No impurity phases are detected in the sample. The single crystal image of Cd_3As_2 with I4_1/acd symmetry is depicted in Figure S3.

We have compared the crystal structure of Cd_3As_2 single crystal samples of I4_1cd and I4_1/acd symmetries (Figure S4) using synchrotron X-ray diffraction technique. It can be seen that the diffraction peaks (marked with star) indexed with I4_1cd space group are more pronounced than for sample with I4_1/acd space group, indicating the higher crystallinity of Cd_3As_2 single crystals in I4_1cd.

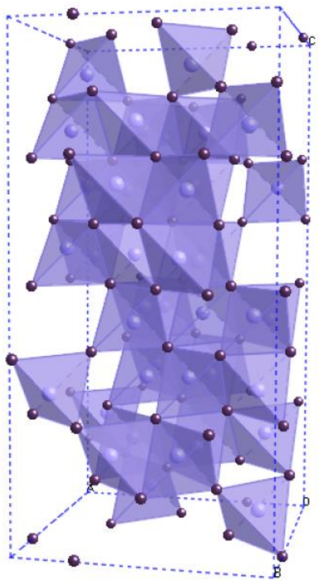

Figure S1. Crystal structure of Cd_3As_2 with I4_1/acd symmetry.

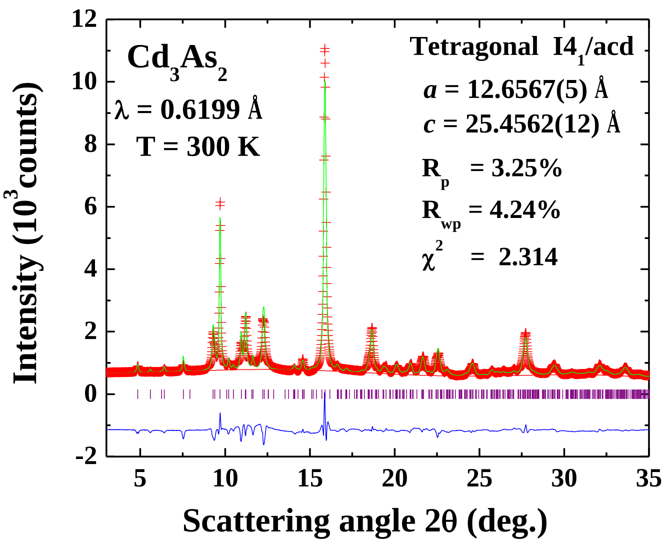

Figure S2. Observed (red cross) and calculated (green color) synchrotron x-ray diffraction pattern of the Cd_3As_2 sample refined with $I4_1/acd$ space group.

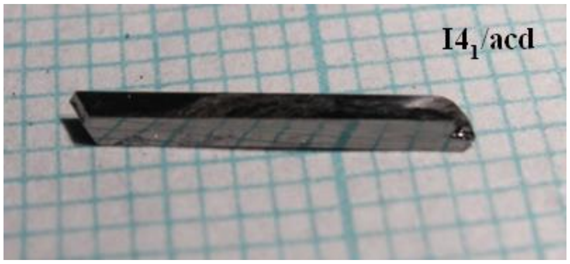

Figure S3. Single crystal image of Cd_3As_2 with $I4_1/acd$ symmetry.

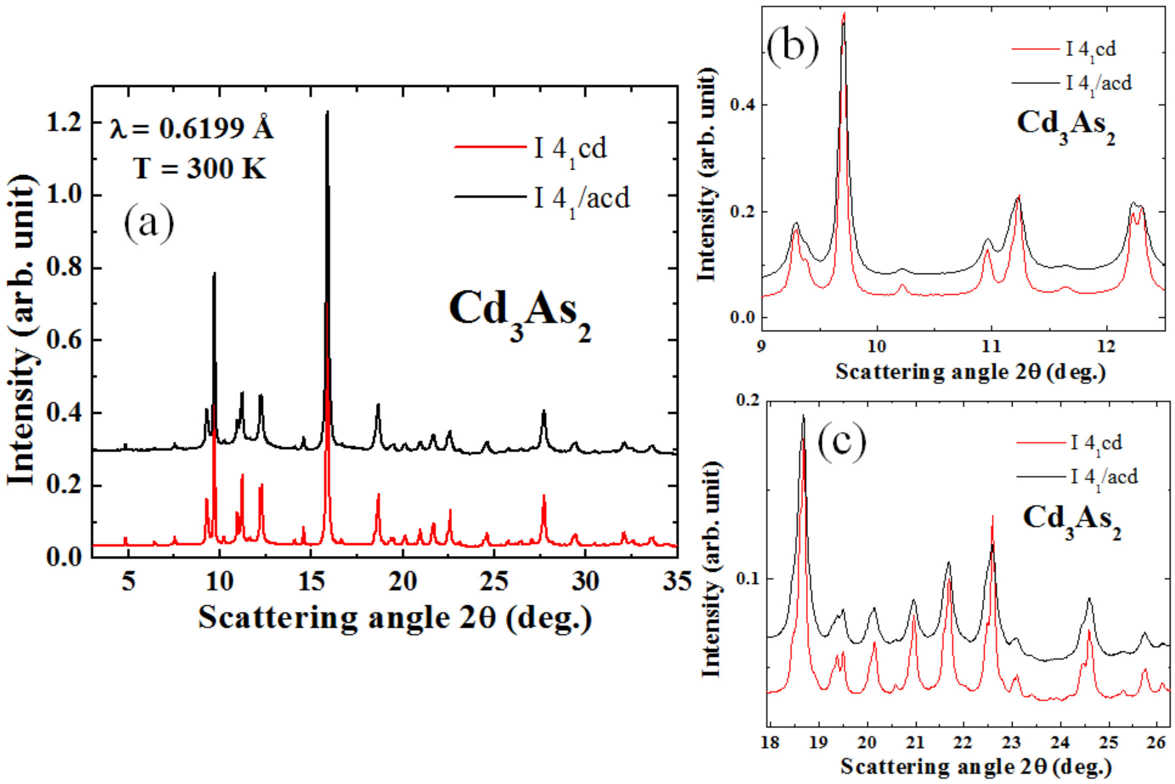

Figure S4. Synchrotron x-ray diffraction patterns of Cd_3As_2 with $I4_1cd$ and $I4_1/acd$ symmetries are compared. The diffraction peaks are more pronounced for $I4_1cd$ than $I4_1/acd$, indicating that Cd_3As_2 of $I4_1cd$ has a higher crystallinity.

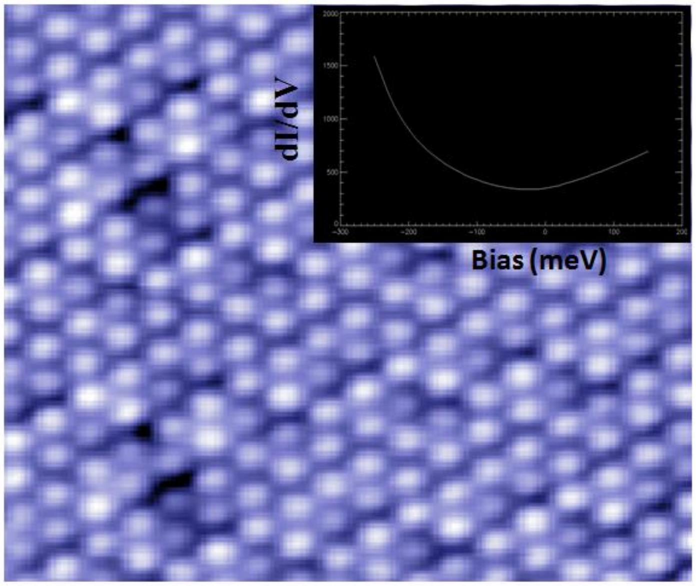

Figure S5. STM topography measurements performed on the cleaved (112) surface of $I4_1/acd$

Cd_3As_2 single crystal.

Table 1. Lists of structural parameters for Cd_3As_2 sample refined with $I4_1/acd$ space group at 300 K, where B_{iso} represents the isotropic temperature parameter and M represents the multiplicity.

Cd_3As_2

Tetragonal $I4_1/acd$ space group (No. 142, Z = 32)

T = 300 K,

$a = b$ = 12.6567(5)Å , c = 25.4562(12) Å

Atom	x	y	z	M	B_{iso}(Å ^{2})	Occupancy
Cd1	0.1386(3)	0.3695(4)	0.0531(4)	*32g*	2.713)	1.000
Cd2	0.1093(4)	0.6445(3)	0.0727(2)	*32g*	4.10(2)	0.991(4)
Cd3	0.1176(3)	0.1043(5)	0.0619(4)	*32g*	1.82(2)	0.996(5)
As1	0.2479(2)	0.2577(4)	0.1244(5)	*32g*	1.56(4)	1.012(3)
As2	0	1/4	0.9991(3)	*16d*	5.42(3)	1.005(4)
As3	1/4	0.5094(3)	0	*16e*	0.63(2)	0.992(6)

χ^2 = 2.314, R_p= 3.25%, R_{wp}= 4.24%
